# Estimation of burden of ischemic heart diseases in Isfahan, Iran, 2014: using incompleteness and misclassification adjustment models

**DOI:** 10.1186/s40200-017-0294-6

**Published:** 2017-03-15

**Authors:** Mehran Shams-Beyranvand, Farshad Farzadfar, Shohreh Naderimagham, Maryam Tirani, Mohammad Reza Maracy

**Affiliations:** 1 Non-Communicable Diseases Research Center, Endocrinology and Metabolism Population Sciences Institute, Tehran University of Medical Sciences, Tehran, Iran; 2 Student Research Center, School of Health, Isfahan University of Medical Sciences, Isfahan, Iran; 3 Department of Epidemiology and Biostatistics, School of Health, Isfahan University of Medical Sciences, St. Hezarjarib, Isfahan, 8174673461 Iran

**Keywords:** Burden of disease, Ischemic heart diseases (IHDs), Disability-adjusted life years (DALYs), Years lived with disability (YLDs), Years of life lost due to premature mortality (YLLs)

## Abstract

**Background:**

Over the past decade, cardiovascular diseases (CVDs) have been the leading cause of death in the world. Ischemic heart diseases (IHDs) are the main form of CVDs and are the most important single cause of death around the world. This study aimed to estimate the burden of IHDs in the city of Isfahan by calculating disability-adjusted life years (DALYs).

**Methods:**

This population-based study was conducted on the population living in the city of Isfahan in 2014. Years of life lost due to premature mortality (YLLs) were calculated by multiplying the number of deaths due to IHDs (that was collected from death registration system (DRS) and was adjusted for incompleteness and misclassification) with life expectancy (that was extracted from the Global Burden of Diseases, Injuries, and Risk Factors study (GBD) 2013). Years lived with disability (YLDs) were calculated by multiplying the prevalent cases of IHDs in each age-sex group (that was calculated using the stepwise approach to non-communicable disease risk factor surveillance (STEPS)) with total disability weight of IHDs (that was calculated using the GBD 2013). DALYs were calculated as the sum of YLDs and YLLs.

**Results:**

In 2014, the number of DALYs due to IHDs was 43517.71 years which was formed of 31891.79 years of YLLs and 11625.92 years of YLDs. The rate of DALYs due to IHDs was 4412.33 (95% uncertainty interval (UI): 3636.70–5162.72) person-years per 100,000 persons in males and 3476.66 (95% UI: 2948.95–4010.51) person-years per 100,000 persons in females. The highest rates of YLLs, YLDs, and DALYs due to IHDs in both sexes were occurred in 80 years and older.

**Conclusions:**

The highest proportion of the burden of IHDs in the city of Isfahan was attributed to YLLs in the elderly people. Since the majority of the population of Isfahan is <60 years old and IHDs are long-lasting, the health authorities are recommended to design and implement educational and cultural programs to inform people about the risk factors and the methods to prevent these diseases. These programs can be used as a strategy to reduce the incidence of IHDs from an early age.

## Background

Mortality indicators alone do not properly reflect the health status of a population. Therefore, the health status of communities is assessed by the summary measures of population health (SMPH) that utilizes a combination of data on mortality and non-fatal consequences of diseases and injuries to express a population’s health status in quantitative form. One of these measures is disability-adjusted life years (DALYs) that indicate the years of life either lost due to premature death or lost as a result of disability caused by non-fatal diseases. The Global Burden of Disease study (GBD) uses this indicator for the calculation of burden of diseases [1].

In the past decade, cardiovascular diseases (CVDs) have been the leading cause of death all over the world [2]. In 2015, CVDs accounted for nearly 17.9 million deaths (32.12% of all deaths) and almost 347.5 million DALYs (14.12% of the total DALYs) in the world [3]. Ischemic heart diseases (IHDs) are the main form of CVDs which lead to 7.2 million deaths each year, most of which occur in developing countries [4]. In 2013, IHDs led to 8.1 million deaths in the world (14.8% of total deaths and almost 50% of deaths due to CVDs) [5]; and in 2015 they accounted for almost 164million DALYs (6.70% of the total DALYs and almost 47.19% of DALYs due to CVDs) in the world. According to the GBD 2015, CVDs were the cause of 20.23% of the total burden of diseases in Iran, of which 10.95% were related to IHDs (the leading cause of the total burden of diseases). In addition, IHDs were the leading cause of death (with 25.82% of all deaths) in 2015 in Iran [3]. In 2014, 46% of all deaths were reported to be due to CVDs in Iran [6]. In 2011, 43.92% of all deaths are caused by CVDs in Isfahan province [7].

To make the right decision about health priority, it is necessary to have some measures such as DALYs [8]. Also, Sub-national studies with more data on provincial and even city data to estimate the burden of IHDs can help to provide objective evidence and to access appropriate and detailed fundamental data which health policy makers need to make health policies, and design and manage appropriate interventions [9].

Since there is not already published information on the burden of IHDs at the sub-national level in Iran, the present study was designed and implemented in order to estimate the burden of IHDs in the city of Isfahan, which is the second largest city in Iran and located in the center of this country.

## Methods

This population-based study was conducted in the city of Isfahan in 2014. The study population was calculated by a demographic projection model, called DemProj, which uses the cohort-component method for making population projection [10]. This method has been described in detail elsewhere [11]. The inputs were the population in the base year (census 2011), the total fertility rate (1.7) that was collected from Isfahan University of Medical Sciences, the sex ratio at birth (102) that was collected from statistical center of Iran, and the life expectancy at birth (73 and 77 years for males and females, respectively) that were collected from NCDRC, Tehran University of Medical Sciences. Also, life table (Coale-Demeny West model) and age- specific fertility rate (average model table) were selected as options in DemProj.

**Fig. 1:**
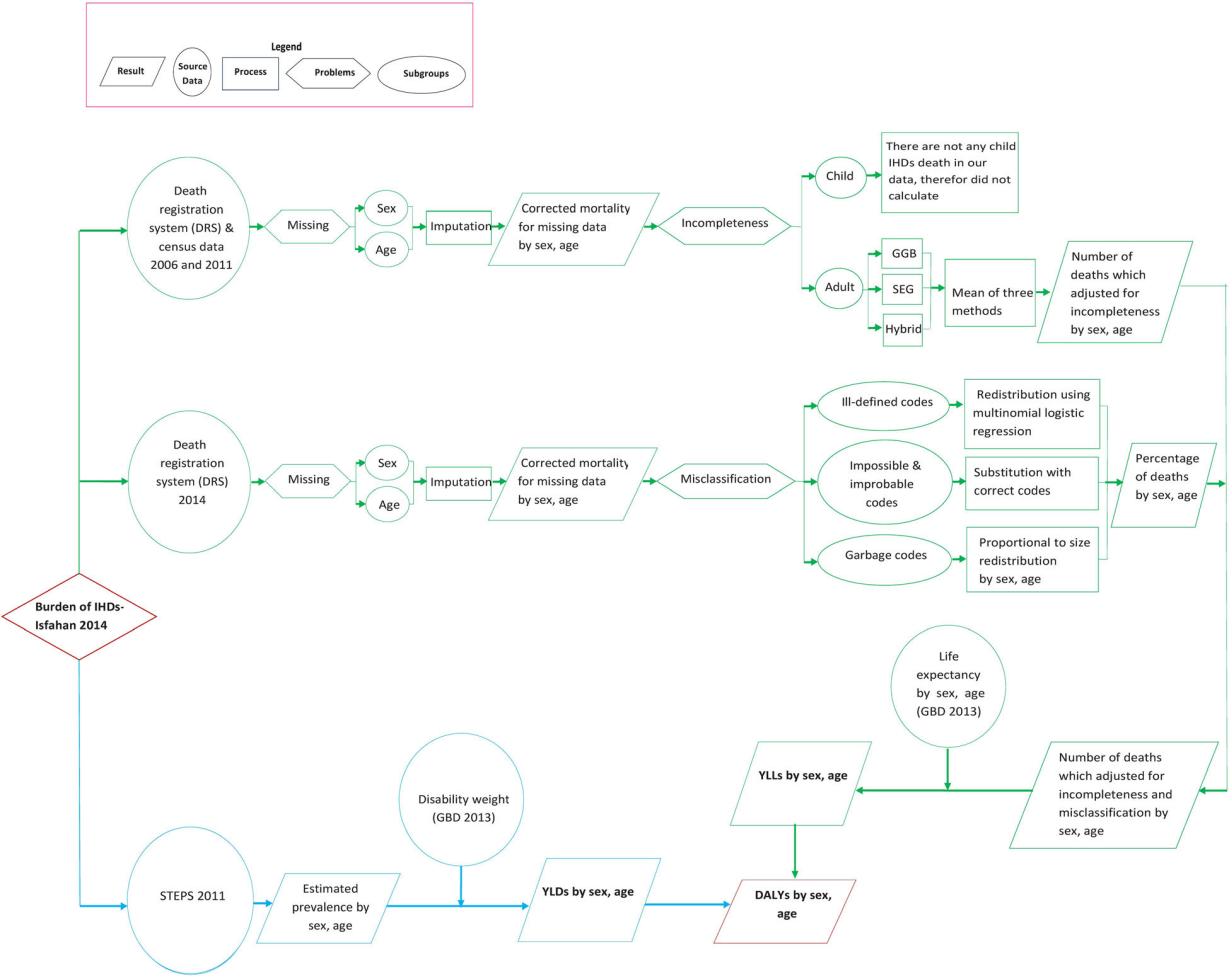
Scientific scheme of the processes, data sources and estimation of Burden of ischemic heart diseases

The burden of IHDs was estimated by calculating DALYs which is formed of two component including years of life lost due to premature mortality (YLLs) and years lived with disability (YLDs) [12]. The scientific scheme of the processes of this study is plotted in Fig. 1.

### Calculating YLLs

In order to calculate YLLs, we need to have two components: 1) the number of deaths due to IHDs that adjusted for incompleteness and misclassification by sex and age (N); 2) life expectancy by sex and age (L). To determine the first component, the four following steps were taken:

In the first step, it was tried to calculate the incompleteness of mortality data among children less than 5 years old and people aged 5 years and older. The first was not calculated because deaths due to IHDs in this age group are not common and in the present study there was not any death due to IHDs in this age group. The latter was calculated through the following stages: a) collecting the population data of two censuses (2006 and 2011); b) collecting mortality data in 2006–2011 that registered based on international statistical classification of diseases and related health problems (ICD-10) codes [13] (that were set in according to the epidemiological conditions in Iran) in Bagh-e-Rezvan cemetery in Isfahan; c) cleaning mortality data; d) selecting the cases of deaths between the two censuses; e) imputing unknown ages (using linear regression model); f) estimating the percentage of completeness of death registration system (DRS) using death distribution methods (generalized growth balance (GGB), synthetic extinct generations (SEG), and a hybrid of the two methods separately); and g) calculating the final percentage of completeness of DRS (the mean of completeness resulted from three death distribution methods) in Isfahan in 2011 as an alternative for the final percentage of completeness of DRS in 2014 [14–17].

In the second step, the following stages were performed to adjust misclassification: a) collecting mortality data in 2014 that registered based on ICD-10 codes in Bagh-e-Rezvan cemetery in Isfahan; b) cleaning mortality data; c) imputing unknown ages (using linear regression model); d) imputing the causes of death without any ICD-10 code, causes of death that were coded as R00-R99 (ill-defined codes), DxC-DxD (cancer without details), F70-F79 (mental retardation), and impossible and improbable age codes with the correct ones (using multinomial logistic regression); e) exchanging impossible and improbable sex codes with the correct ones (by reviewing the name of the deceased persons); f) collapsing mortality data by age, sex, and cause of death; g) redistributing garbage codes that are including A40-A41 (septicemia), I26 (pulmonary embolism), I50 (heart failure), I74 (arterial embolism and thrombosis), and N17 (Acute kidney failure) among equivalent correct ICD-10 codes in every age and sex group (proportional to size according to number of deaths); and h) calculating the percentage of deaths due to IHDs among people aged 5 years and older by age and sex in 2014.

In the third step, the total number of adjusted deaths for incompleteness among people aged 5 years and older by age and sex in 2014 was calculated through the equation [14]

$$ total\  number\  o f\  adjusted\  deaths\  f o r\  in completeness=\frac{total\  number\  o f\  deaths\  in\ 2014}{final\  percentage\  o f\  completeness\  o f\  DRS\  in\ 2011} $$

In the fourth step, the number of deaths due to IHDs by age and sex in 2014 which adjusted for incompleteness and misclassification (N) was calculated through the equation

$$ N=\left(\frac{total\  number\  o f\  deaths\  in\ 2014}{final\  percentage\  o f\  completeness\  o f\  DRS\  in\ 2011}\right) \times percentage\  o f\  deaths\  due\  t o\  IHDs\  in\ 2014 $$

Uncertainty intervals (UI) for N was calculated through the equation

$$ {N}_{\left( lower\  boundary, \kern0.75em  upper\  boundary\right)}=\left(\frac{total\  number\  o f\  deaths\  in\ 2014}{final\  percentage\  o f\  completeness\  o f\  DRS\  in\ 2011 \pm 0.19}\right)\times percentage\  o f\  deaths\  due\  t o\  IHDs\  in\kern0.5em 2014 $$

; Where 0.19 is the standard deviation of the final percentage of completeness of DRS that is equal to 20% of the final percentage of completeness of DRS [16].

To determine the second component of YLLs i.e. life expectancy (L), we used the standard abridged life table which had been used in the GBD 2010 and GBD 2013 [17].

Finally, we used the equation *YLLs* = *N* × *L* to calculate YLLs due to IHDs.

UI for YLLs was calculated through multiplying the lower and upper boundaries of N by L.

### Calculating YLDs

We need to have the number of prevalent cases (P) and disability weight (DW) of IHDs, in order to calculate YLDs. The number of prevalent cases were calculated using the stepwise approach to non-communicable disease risk factor surveillance (STEPS) study that was conducted in Isfahan province in 2011 [18]. The STEPS study methodology has been described elsewhere [19].

In this study, we calculated total disability weight of IHDs as follows: a) the number of every sequela of IHDs and its subsets plus disability weights of subsets were extracted from the GBD 2013; b) the percentage of subsets of every sequela of IHDs in 2013 was calculated by dividing the number of every subset by the number of every sequela; c) the percentage of every sequela of IHDs in 2013 was calculated by dividing the number of every sequela of IHDs by the total number of all sequelae of IHDs; d) disability weight of every sequela of IHDs in 2013 was calculated by multiplying the percentage of every subset of every sequela of IHDs by disability weight of that subset and then, summing up all the subsets; e) total disability weight of IHDs in 2013 in the world was calculated by multiplying the percentage of every sequela of IHDs by disability weight of the same sequela, and then, summing up all the sequelae of IHDs.

Then, YLDs due to IHDs were calculated through the equation *YLDs* = *P* × *DW*; Where P is the number of prevalent cases of IHDs in each age-sex group, DW is total disability weight of IHDs in GBD 2013.

UI for the number of YLDs was calculated through the equation

*YLDs*_(*lower boundary* ,  *upper boundary*)_ = (*P* ± *SE*(*P*) × 1.96) × *DW*; Where SE(p) is the standard error of the number of prevalent cases that were calculated using the STEPS study.

While estimating the burden of diseases using statistical models, two types of uncertainty may occur: uncertainty arising from sampling (sample uncertainty) and uncertainty resulting from the use of statistical models (model uncertainty) [15]. In order to calculate “sample uncertainty” related to the number of YLLs and YLDs we used a simulation model to take 1000 draws from the number of YLLs and YLDs for each age, sex and then the 95% UI was calculated as the 2.5th and 97.5th percentiles of the 1000 draws. In this study, because there were not proper computing facilities, “model uncertainty” of the number of YLLs and YLDs were not determined.

### Calculating DALYs

Finally, DALYs due to IHDs in Isfahan in 2014 was calculated through the equation *DALYs* = *YLLs* + *YLDs* in each age-sex group.

Data analysis was performed using STATA software version 12.0 (Stata Corp, College Station, TX, USA), R statistical software version 3.1.0 (R Foundation for Statistical Computing, Vienna, Austria), Spectrum software version 5.03 (Avenir Health, Washington, USA), and Excel 2010 software (Microsoft Corp, Redmond, Washington, USA).

## Results

In 2014, Isfahan city population (in age groups that were used in this study) was estimated to be 1,100,793 people, of whom 560,764 (50.94%) were males and 540,029 (49.06%) were females (Table 1).

**Table 1:** The number of adjusted deaths due to ischemic heart diseases after redistribution by age, sex

Age	Population (1)	Total number of deaths before redistribution in 2014 (2)	Total number of adjusted deaths for incompleteness before redistribution in 2014 (3) ((2)/0. 95^a^)	Total number of deaths after redistribution in 2014 (4)	Total number of deaths due to IHDs after redistribution in 2014 (5)	Percentage of deaths due to IHDs after redistribution in 2014 (%) (6) ((5)/(4))	Number of adjusted deaths due to IHDs after redistribution in 2014 (7) ((3) × (6))	Life expectancy (GBD 2013) (8)
Female								
25–29	113083	23	24.21	23.22	2.11	9.09	2.20	61.40
30–34	84388	25	26.32	23.22	2.11	9.09	2.39	54.46
35–39	68341	38	40.00	33.77	4.82	14.27	5.71	51.53
40–44	64163	32	33.68	33.78	5.28	15.63	5.26	46.64
45–49	55959	41	43.16	41.15	9.04	21.97	9.48	41.80
50–54	46931	55	57.89	52.74	13.41	25.43	14.72	37.05
55–59	34669	81	85.26	83.30	21.65	25.99	22.16	32.38
60–64	23496	116	122.11	119.08	43.61	36.62	44.72	27.81
65–69	17314	152	160.00	140.01	58.61	41.86	66.98	23.29
70–74	12898	166	174.74	161.80	56.68	35.03	61.21	18.93
75–79	10025	231	243.16	214.54	101.57	47.34	115.11	14.80
> =80	8762	870	915.79	883.78	387.64	43.86	401.67	10.99
total	540029	1830	1926.32	1810.39	706.53	39.03	751.61	-
Male								
25–29	111437	58	61.05	58.19	1.56	2.68	1.64	61.40
30–34	86075	83	87.37	82.09	7.27	8.86	7.74	54.46
35–39	71916	62	65.26	67.49	4.43	6.56	4.28	51.53
40–44	67554	73	76.84	75.82	9.35	12.33	9.47	46.64
45–49	58635	92	96.84	95.57	28.81	30.15	29.20	41.80
50–54	49678	150	157.89	152.65	46.77	30.64	48.38	37.05
55–59	37826	207	217.89	193.16	69.33	35.89	78.20	32.38
60–64	25616	213	224.21	211.54	70.27	33.22	74.48	27.81
65–69	19142	249	262.11	236.33	92.14	38.99	102.20	23.29
70–74	14556	259	272.63	259.79	118.91	45.77	124.78	18.93
75–79	10396	302	317.89	313.83	116.79	37.21	118.29	14.80
> =80	7933	869	914.74	895.87	361.23	40.32	368.82	10.99
total	560764	2617	2754.72	2642.33	926.86	35.08	967.48	-
Total	1100793	4447	4681.04	4452.72	1633.39	36.68	1719.09	-

^a^The final percentage of completeness of death registration system in 2011 that obtained from death distribution methods

The percentage of completeness of DRS which was estimated using GGB, SEG, and hybrid methods respectively, were 92%, 100%, and 92%. The mean of completeness resulted from three death distribution methods was 95% which was considered as the final percentage of completeness of DRS in Isfahan in 2011. Thus the final percentage of incompleteness was 5%.

The number of registered deaths in Bagh-e-Rezvan cemetery during the year 2014 was 4741 which was changed to 4625 after data cleaning. Moreover, 498 ICD-10 codes were imputed, of which 257 deaths were without ICD-10 code, 205 deaths were coded as R00-R99, 33 deaths were coded as DxC-DxD, two deaths were coded as F70-F79, and one death was coded as impossible and improbable age death. On the other hand, there was only one impossible and improbable sex code that was corrected via reviewing the name of the deceased person. In total, there were five garbage codes (including A40-A41, I26, I50, I74, and N17) which included a total of 828 people; they were redistributed among the correct ICD-10 codes of the same age-sex group.

After redistribution, 36.7% of deaths were attributed to IHDs and the number of simulated deaths due to IHDs was 1719, of which 967 (56.3%) occurred among males and 752 (43.7%) occurred among females. The highest numbers of deaths were observed among males and females aged 80 years and older (372 and 401, respectively) and the lowest numbers of deaths were observed among males and females aged 25–29 years old (two and two, respectively) (Table 1).

The highest and lowest prevalent cases of IHDs in males were observed in 25–29 years old and 80 years and older people, respectively, while in females, 55–59 and 25–29 years old people had the highest and lowest prevalent cases of IHDs (Table 2).

**Table 2:** The number of prevalent cases of ischemic heart diseases by age, sex in Isfahan city, 2014

	Prevalent cases of IHDs [95% UI]	
Age	Male	Female
25–29	10140.77 [8879.98–11401.55]	2940.16 [2366.10–3514.21]
30–34	5250.58 [4251.83–6249.32]	8185.64 [7110.53–9260.74]
35–39	6975.85 [6133.16–7818.54]	3963.78 [3283.44–4644.12]
40–44	1486.19 [1188.27–1784.10]	3978.11 [3222.29–4733.92]
45–49	6039.41 [5328.66–6750.15]	3861.17 [3261.22–4461.12]
50–54	8693.65 [7792.01–9595.29]	7978.27 [7223.99–8732.55]
55–59	4463.47 [4027.53–4899.41]	8216.55 [7638.97–8794.14]
60–64	3714.32 [3372.91–4055.73]	6061.97 [5729.47–6394.46]
65–69	1091.09 [884.37–1297.82]	4155.36 [3901.86–4408.86]
70–74	1732.16 [1541.03–1923.30]	3753.32 [3528.62–3978.02]
75–79	1268.31 [1129.70–1406.92]	3248.10 [3062.76–3433.44]
> =80	1023.36 [913.58–1133.13]	3566.13 [3380.78–3751.49]
total	61684.04 [54649.01–68719.07]	105305.66 [97568.12–113043.19]

The numbers (rates) of DALYs due to IHDs were 24742.74 (95% UI: 20393.29–28950.69) years (4412.33 (95% UI: 3636.70–5162.72) person-years per 100,000 persons) in males and 18774.97 (95% UI: 15925.19–21657.92) years (3476.66 (95% UI: 2948.95–4010.51) person-years per 100,000 persons) in females. In addition, the number (rate) of DALYs was 43517.71 (95% UI: 38212.10–48778.92) years (3953.31 (95% UI: 3471.33–4431.25) person-years per 100,000 persons) in both sexes. The highest and lowest numbers and rates of DALYs in males were observed in 80 years and older and 40–44 years old people, respectively. The highest and lowest numbers and rates of DALYs in females were observed in 80 years and older and 25–29 years old people, respectively. The highest and lowest numbers of DALYs in both sexes were observed in 80 years and older and 40–44 years old people, respectively; moreover, the highest and lowest rates of DALYs in both sexes were observed in 80 years and older and 25–29 years old people, respectively. The number of DALYs were in high proportion at adults <60 years old (45.29% of the number of DALYs lost in males <60 years old and 34.79% of the number of DALYs lost in females <60 years old). The number of YLLs and YLDs were accounted for roughly 73% and 27% of the total number of DALYs due to IHDs in both sexes, respectively. Also, 52.15% of the total number of DALYs due to IHDs in both sexes was attributed to YLLs in elderly (>60 years old) people. The highest rates of YLLs and YLDs due to IHDs in both sexes were occurred in 80 years and older and the lowest rates of YLLs and YLDs due to IHDs in both sexes were occurred in 25–29 years old and 40–44 years old people, respectively (Table 3).

**Table 3:** YLLs, YLDs, and DALYs number and rate of ischemic heart diseases by age, sex

	Male		Female		Both sexes	
Age	Number of YLLs [95% UI]	Rates of YLLs (per 100000) [95% UI]	Number of YLLs [95% UI]	Rates of YLLs (per 100000) [95% UI]	Number of YLLs [95% UI]	Rates of YLLs (per 100000) [95% UI]
25–29	100.46 [83.72–125.58]	90.15 [75.13–112.69]	135.13 [112.60–168.91]	119.49 [99.58–149.37]	235.59 [197.97–269.70]	104.93 [88.17–120.12]
30–34	421.57 [351.31–526.96]	489.77 [408.14–612.21]	130.27 [108.56–162.84]	154.38 [128.65–192.97]	551.84 [448.79–640.88]	323.73 [263.28–375.97]
35–39	220.61 [183.84–275.77]	306.77 [255.64–383.46]	294.13 [245.11–367.67]	430.39 [358.66–537.99]	514.75 [443.10–590.16]	367.00 [315.92–420.77]
40–44	441.90 [368.25–552.37]	654.14 [545.12–817.67]	245.55 [204.63–306.94]	382.70 [318.92–478.38]	687.45 [575.25–788.46]	521.91 [436.73–598.60]
45–49	1220.47 [1017.06–1525.59]	2081.47 [1734.56–2601.84]	396.34 [330.28–495.42]	708.27 [590.22–885.33]	1616.81 [1351.98–1888.52]	1410.90 [1179.80–1648.01]
50–54	1792.44 [1493.70–2240.55]	3608.12 [3006.76–4510.15]	545.47 [454.56–681.84]	1162.29 [968.57–1452.86]	2337.91 [1943.21–2736.43]	2419.97 [2011.42–2832.48]
55–59	2532.20 [2110.16–3165.24]	6694.32 [5578.60–8367.90]	717.54 [597.95–896.92]	2069.68 [1724.73–2587.10]	3249.73 [2672.49–3814.17]	4482.70 [3686.45–5261.28]
60–64	2071.37 [1726.14–2589.21]	8086.22 [6738.51–10107.77]	1243.52 [1036.27–1554.40]	5292.49 [4410.41–6615.61]	3314.89 [2816.51–3825.47]	6749.65 [5734.86–7789.28]
65–69	2380.12 [1983.43–2975.15]	12434.01 [10361.67–15542.51]	1559.87 [1299.89–1949.84]	9009.31 [7507.75–11261.63]	3939.99 [3357.90–4539.67]	10807.52 [9210.82–12452.47]
70–74	2362.15 [1968.46–2952.69]	16228.03 [13523.35–20285.03]	1158.71 [965.59–1448.39]	8983.65 [7486.38–11229.56]	3520.86 [2946.31–4077.05]	12824.59 [10731.80–14850.46]
75–79	1750.67 [1458.89–2188.34]	16839.86 [14033.22–21049.83]	1703.64 [1419.70–2129.55]	16993.94 [14161.61–21242.42]	3454.31 [2938.76–3947.37]	16915.50 [14390.85–19329.94]
> =80	4053.35 [3377.79–5066.69]	51094.82 [42579.02–63868.54]	4414.30 [3678.58–5517.88]	50380.07 [41983.39–62975.08]	8467.65 [7248.68–9706.83]	50719.70 [43418.28–58142.13]
total	19347.31 [16122.75–24184.13]	3450.17 [2875.14–4312.71]	12544.48 [10453.74–15680.60]	2322.93 [1935.77–2903.66]	31891.79 [26940.92–36824.71]	2897.16 [2447.41–3345.29]
	Number of YLDs [95% UI]	Rates of YLDs (per 100000) [95% UI]	Number of YLDs [95% UI]	Rates of YLDs (per 100000) [95% UI]	Number of YLDs [95% UI]	Rates of YLDs (per 100000) [95% UI]
25–29	1054.64 [923.52–1185.76]	946.40 [828.74–1064.07]	305.78 [246.07–365.48]	270.40 [217.61–323.19]	1360.42 [1212.37–1516.19]	605.92 [539.98–675.30]
30–34	546.06 [442.19–649.93]	634.40 [513.73–755.07]	851.31 [739.50–963.12]	1008.80 [876.30–1141.30]	1397.37 [1259.23–1558.69]	819.75 [738.71–914.38]
35–39	725.49 [637.85–813.13]	1008.80 [886.94–1130.66]	412.23 [341.48–482.99]	603.20 [499.67–706.73]	1137.72 [1018.64–1258.05]	811.17 [726.27–896.96]
40–44	154.56 [123.58–185.55]	228.80 [182.94–274.66]	413.72 [335.12–492.33]	644.80 [522.29–767.31]	568.29 [482.20–651.61]	431.45 [366.09–494.71]
45–49	628.10 [554.18–702.02]	1071.20 [945.14–1197.26]	401.56 [339.17–463.96]	717.60 [606.10–829.10]	1029.66 [925.19–1127.10]	898.53 [807.36–983.56]
50–54	904.14 [810.37–997.91]	1820.00 [1631.24–2008.76]	829.74 [751.30–908.18]	1768.00 [1600.85–1935.15]	1733.88 [1605.29–1852.03]	1794.74 [1661.63–1917.04]
55–59	464.20 [418.86–509.54]	1227.20 [1107.34–1347.06]	854.52 [794.45–914.59]	2464.80 [2291.54–2638.06]	1318.72 [1240.25–1391.66]	1819.05 [1710.80–1919.66]
60–64	386.29 [350.78–421.80]	1508.00 [1369.39–1646.61]	630.44 [595.87–665.02]	2683.20 [2536.03–2830.37]	1016.73 [965.73–1068.90]	2070.24 [1966.39–2176.45]
65–69	113.47 [91.97–134.97]	592.80 [480.48–705.12]	432.16 [405.79–458.52]	2496.00 [2343.73–2648.27]	545.63 [511.13–579.70]	1496.68 [1402.04–1590.14]
70–74	180.15 [160.27–200.02]	1237.60 [1101.04–1374.16]	390.35 [366.98–413.71]	3026.40 [2845.22–3207.58]	570.49 [539.24–601.49]	2077.99 [1964.15–2190.91]
75–79	131.90 [117.49–146.32]	1268.80 [1130.14–1407.46]	337.80 [318.53–357.08]	3369.60 [3177.33–3561.87]	469.71 [445.80–492.14]	2300.12 [2183.05–2409.97]
> =80	106.43 [95.01–117.85]	1341.60 [1197.69–1485.51]	370.88 [351.60–390.15]	4232.80 [4012.80–4452.80]	477.31 [455.76–499.42]	2858.98 [2729.89–2991.44]
total	5395.43 [4726.08–6064.79]	962.16 [842.79-1081.52]	6230.49 [5585.85–6875.13]	1153.73 [1034.36–1273.10]	11625.92 [10660.82–12596.97]	1056.14 [968.47–1144.35]
	Number of DALYs [95% UI]	Rates of DALYs (per 100000) [95% UI]	Number of DALYs [95% UI]	Rates of DALYs (per 100000) [95% UI]	Number of DALYs [95% UI]	Rates of DALYs (per 100000) [95% UI]
25–29	1155.10 [1024.35–1298.07]	1036.55 [919.22–1164.84]	440.90 [374.68–505.52]	389.89 [331.33–447.03]	1596.00 [1437.15–1747.43]	710.85 [640.10–778.30]
30–34	967.63 [832.35–1115.03]	1124.17 [967.00–1295.41]	981.58 [867.22–1099.67]	1163.18 [1027.66–1303.11]	1949.21 [1762.64–2149.70]	1143.48 [1034.03–1261.09]
35–39	946.10 [846.72–1043.17]	1315.57 [1177.37–1450.54]	706.37 [616.62–800.19]	1033.59 [902.26–1170.88]	1652.47 [1513.31–1792.52]	1178.17 [1078.95–1278.03]
40–44	596.46 [489.36–695.78]	882.94 [724.40–1029.96]	659.28 [566.91–746.41]	1027.50 [883.54–1163.30]	1255.74 [1119.77–1384.73]	953.36 [850.13–1051.29]
45–49	1848.57 [1589.23–2108.88]	3152.67 [2710.38–3596.62]	797.90 [692.79–897.49]	1425.87 [1238.03–1603.83]	2646.47 [2350.36–2921.31]	2309.43 [2051.03–2549.27]
50–54	2696.58 [2302.64–3063.82]	5428.12 [4635.13–6167.35]	1375.21 [1229.67–1513.51]	2930.29 [2620.17–3224.97]	4071.79 [3663.75–4482.93]	4214.71 [3792.35–4640.28]
55–59	2996.40 [2423.70–3530.31]	7921.52 [6407.49–9333.02]	1572.06 [1412.12–1733.34]	4534.48 [4073.15–4999.68]	4568.45 [3982.08–5125.59]	6301.75 [5492.91–7070.27]
60–64	2457.65 [2019.57–2879.57]	9594.22 [7884.03–11241.30]	1873.97 [1619.39–2135.13]	7975.69 [6892.19–9087.22]	4331.62 [3839.37–4847.75]	8819.88 [7817.58–9870.81]
65–69	2493.59 [2008.35–2999.36]	13026.81 [10491.82–15668.97]	1992.03 [1675.71–2331.29]	11505.30 [9678.32–13464.75]	4485.62 [3898.86–5095.85]	12304.20 [10694.71–13978.08]
70–74	2542.30 [2043.83–3014.13]	17465.62 [14041.12–20707.16]	1549.06 [1307.41–1775.99]	12010.05 [10136.50–13769.47]	4091.35 [3516.84–4641.14]	14902.57 [12809.92–16905.15]
75–79	1882.58 [1509.81–2232.27]	18108.66 [14522.95–21472.35]	2041.44 [1692.31–2402.20]	20363.54 [16880.93–23962.07]	3924.02 [3397.42–4406.87]	19215.62 [16636.87–21580.08]
> =80	4159.78 [3303.39–4970.33]	52436.43 [41641.11–62653.80]	4785.18 [3870.37–5717.20]	54612.86 [44172.26–65249.92]	8944.96 [7730.57–10183.09]	53578.68 [46304.71–60994.85]
total	24742.74 [20393.29–28950.69]	4412.33 [3636.70–5162.72]	18774.97 [15925.19–21657.92]	3476.66 [2948.95–4010.51]	43517.71 [38212.10–48778.92]	3953.31 [3471.33–4431.25]

**Fig. 2:**
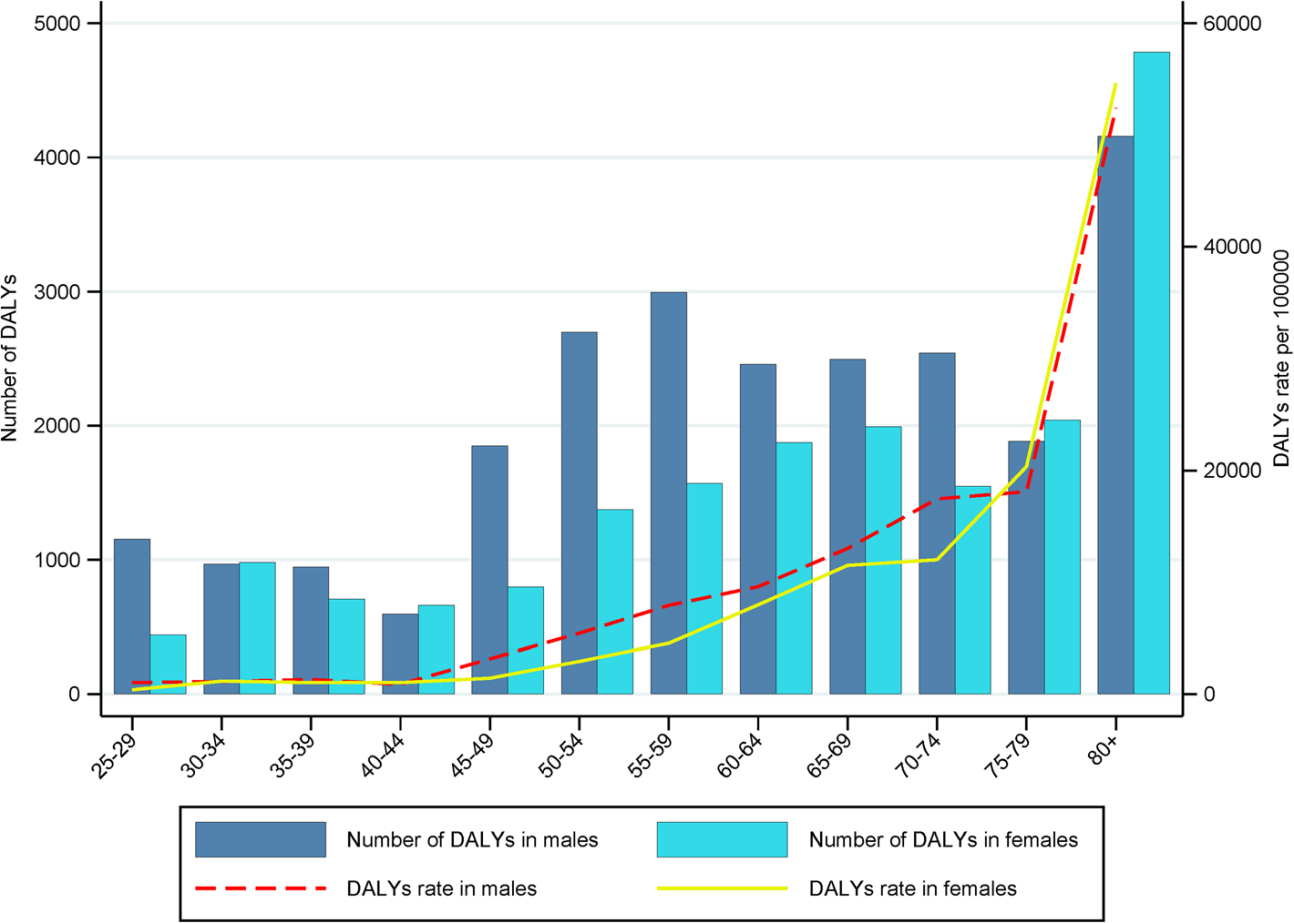
Disability-adjusted life years due to ischemic heart diseases by age and sex in Isfahan city, 2014

The rates of DALYs were increased with increasing the age in both sexes and were higher in males than in females in all age groups, except in 30–34, 40–44, 75–79 years old people, and 80 years and older (Fig. 2).

Total disability weight of IHDs that was calculated using data from GBD 2013 and used in this study was 0.104 (Table 4).

**Table 4:** Calculation of total disability weight of ischemic heart diseases in the world in 2013 [29]

	Number of every sequela and its subsets (thousands-GBD 2013)	Disability weight of subsets of every sequela (GBD 2013)	Percentage of every subset of every sequela	Percentage of every sequela	Disability weight of every sequela	Total disability weight of IHDs
Ischemic heart diseases	-	-	-	-	-	0.104
AMI SEQUELA	-	-	-	0.01	0.099	-
Acute myocardial infarction (First 2 days)	29.70	0.432	7%	-	-	-
Acute myocardial infarction (3 to 28 days)	385.90	0.074	93%	-	-	-
ANGINA SEQUELA	-	-	-	0.99	0.104	-
Mild	13157.00	0.033	35%	-	-	-
Moderate	6914.00	0.08	18%	-	-	-
Severe	17945.60	0.167	47%	-	-	-
total	38432.20	-	-	-	-	-

## Discussion

Based on our findings, the rates of DALYs due to IHDs in the city of Isfahan in 2014 were increased in males and females with increasing age and the highest rate of burden of IHDs was seen in the elderly people. Also, IHDs were the leading cause of death in the city of Isfahan in 2014. This finding is consistent with the results of the GBD 2015 in the world and Iran [3] and other studies [20, 21].

In this study, notable gender differences were observed for the total numbers and rates of YLLs and DALYs due to IHDs, for which the total numbers and rates of YLLs and DALYs were higher in males than females. The same results have been observed in other studies [3, 5, 9, 22]. For the Probable reason, females may be more sensitive to health knowledge, having better behavior to seek health care and having better access to primary prevention [23].

In addition based on the findings of the present study, about one-half of the number of DALYs due to IHDs in males and one-third of the number of DALYs due to IHDs in females were occurred in <60 years old. Also, the percentage of the population in males and females <60 years old were 86.15% and 86.57% respectively. These results show that the burden of IHDs was more in people who known as productive persons and it will affect national economies [24] and also emphasize the importance of primary preventive measures for CVDs even in the younger people. By accepting the increase in the burden of IHDs with increasing age, primary preventive measures supplemented with secondary and tertiary prevention measures should be adapted to each population group and maintained up to older ages [25]. In 2014, Moran et al. indicated that in the North Africa/Middle East and South Asia regions—regions with high IHDs burden— about one-third (29%) of the number of DALYs in males and a quarter (24%) of the number of DALYs in females <50 years old were due to IHDs [24].

Also, GBD 2015 showed that IHDs are the leading cause of burden of diseases in the Middle East and North African countries [3]. The high burden of IHDs in these countries is probably attributed to several factors such as increasing of the prevalence of risk factors, limited access to health care services in both prevention and treatment of IHDs. Thus, these countries may need to emphasize health promotion and disease prevention, control of modifiable risk factors, and treatment of acute and chronic medical conditions to reduce the burden of IHDs [26].

The present study showed that the total numbers and rates of YLDs due to IHDs were lower in males than females. It is probably due to the lower prevalent cases of IHDs in males. These results are inconsistent with the results of the GBD 2015 in the world and Iran [3] and another study [9]. This inconsistency might be due to the differences between our study and other studies in terms of the sources of data and methods that we used to estimate the prevalent cases and disability weights. Estimations of the prevalent cases in the GBD 2010 and GBD 2013 were carried out based on a systematic review and then using Bayesian meta-regression method [12], while, we used the data that were collected by STEPS study in the present study.

It is worth to mention that the data which is from GBD studies are representative at the national level. Having the distribution of diseases at the sub-national level would be a clue for performing the interventions that reduce or control main health problems among sub-national populations [27]. Thus, it is recommended to conduct further studies to estimate the burden of diseases at subnational levels to prioritize health issues.

The strengths of this study are the adjustment of incompleteness and misclassification of mortality data, calculation of “sample uncertainty” and thus, reducing the measurement error, and use of the data that collected by the STEPS study to estimate the prevalent cases of IHDs.

This study has several limitations. Firstly, since the DRS did not provide the data on every type of IHDs separately, it was not possible to estimate the burden of every type of IHDs separately. Secondly, the validity of our findings may be affected by the quality of death certificates which are subject to information bias probably due to differences in physicians’ education programs and also their training in completing death certificates [28]. Thirdly, in order to calculate the incompleteness of mortality data in DRS on 5 years old and above people, we had to calculate the incompleteness of mortality data in 2011 and used it as an alternative for the year 2014. Fourthly, it was not possible to calculate directly the prevalent cases of IHDs since the mild cases of IHDs (who had not been hospitalized) were not registered. In addition, the cases that referred to hospitals in other cities and districts were possibly not registered as well. As a result, the prevalent cases of these diseases were estimated using data from the STEPS study. Fifthly, because of the lack of data for all sequelae we did not consider comorbidity in the calculation of disability weight in this study. Finally, because there were not proper computing facilities, we could not calculate “model uncertainty”.

## Conclusions

The results of this study showed that the highest proportion of burden of IHDs in the city of Isfahan was attributed to YLLs in the elderly people. Since the majority of the population of Isfahan is <60 years old and IHDs are long-lasting, the health authorities are recommended to design and implement educational and cultural programs to inform people about the risk factors and the methods to prevent these diseases. These programs can be used as a strategy to reduce the incidence of these diseases from an early age. Accordingly, it is recommended to train physicians about the correct methods of completing death certificates to reduce information bias in the data, and conduct further similar researches in other cities after the 2016 census to achieve a more accurate estimate on the incompleteness of mortality data among people aged over 5 years, and estimate “model uncertainty” in future studies.

## Abbreviations

CVDs: Cardiovascular diseases
DALYs: Disability-adjusted life years
DRS: Death registration system
DW: Disability weight
GBD: Global burden of diseases, injuries, and risk factors study
GGB: Generalized growth balance
ICD-10: International statistical classification of diseases and related health problems
IHDs: Ischemic heart diseases
NCDRC: Non-communicable Diseases Research Center
SEG: Synthetic extinct generations
SMPH: Summary measures of population health
STEPS: Stepwise approach to non-communicable disease risk factor surveillance
UI: Uncertainty interval
YLDs: Years lived with disability
YLLs: Years of life lost due to premature mortality
